# High Laccase Expression by *Trametes versicolor* in a Simulated Textile Effluent with Different Carbon Sources and PHs

**DOI:** 10.3390/ijerph13080778

**Published:** 2016-08-02

**Authors:** Cristiane Ottoni, Marta F. Simões, Sara Fernandes, Cledir R. Santos, Nelson Lima

**Affiliations:** 1 Biosciences Institute, São Paulo State University—UNESP, Coastal Campus, São Vicente, São Paulo 11330-900, Brazil; c.a.ottoni@hotmail.com; 2 Biology Department, Edge Hill University, St. Helens Road, Lancashire, Ormskirk L39 4QP, UK; simoesm@edgehill.ac.uk; 3 Richmond Pharmacology Ltd., St. George’s University London, Cranmer Terrace, London SW17 0RE, UK; sarafernandes83@gmail.com; 4 Department of Chemical Sciences and Natural Resources, Centro de Excelencia en Investigación Biotecnológica Aplicada al Medio Ambiente (CIBAMA), Scientific and Technological Bioresource Nucleus (BIOREN), Universidad de La Frontera, Temuco 4811-230, Chile; 5 CEB-Centre of Biological Engineering, Micoteca da Universidade do Minho, University of Minho, Campus de Gualtar, Braga 4710-057, Portugal

**Keywords:** alkaline conditions, fixed-bed bioreactor, glycerol, Reactive Black 5, reverse transcriptase -PCR, white-rot fungus

## Abstract

Textile effluents are highly polluting and have variable and complex compositions. They can be extremely complex, with high salt concentrations and alkaline pHs. A fixed-bed bioreactor was used in the present study to simulate a textile effluent treatment, where the white-rot fungus, *Trametes versicolor*, efficiently decolourised the azo dye Reactive Black 5 over 28 days. This occurred under high alkaline conditions, which is unusual, but advantageous, for successful decolourisation processes. Active dye decolourisation was maintained by operation in continuous culture. Colour was eliminated during the course of operation and maximum laccase (Lcc) activity (80.2 U∙L^−1^) was detected after glycerol addition to the bioreactor. *Lcc2* gene expression was evaluated with different carbon sources and pH values based on reverse transcriptase-PCR (polymerase chain reaction). Glycerol was shown to promote the highest *lcc2* expression at pH 5.5, followed by sucrose and then glucose. The highest levels of expression occurred between three and four days, which corroborate the maximum Lcc activity observed for sucrose and glycerol on the bioreactor. These results give new insights into the use of *T. versicolor* in textile dye wastewater treatment with high pHs.

## 1. Introduction

Laccase (Lcc—benzenediol: oxygen oxidoreductase, EC 1.10.3.2) is a ubiquitous enzyme in nature and is widely found in higher plants, fungi, bacteria, insects and lichens [1]. Lcc attracts high interest from environmental biotechnology and industry due to its wide substrate specificity, its high versatility, and its use of molecular oxygen as final electron acceptor [2]. Currently, most studies are focused on production of Lcc by filamentous fungi. The widespread occurrence of highly active Lcc in white-rot fungi (WRF) encourages the search for new sources of these enzymes from these organisms. Lcc can be used for several industrial applications, such as pulp bleaching in the paper industry [3,4], textile dye decolourisation [5,6], and detoxification of environmental pollutants [3,7,8].

The use of Lcc in textile industry has been increasingly very fast due to its ability to bleach textiles, and biological treatments involving Lcc seems to be an attractive solution mainly because most existing treatments (e.g., coagulation/flocculation, adsorption, ion exchange, and electrochemical methods) of dye wastewater utilize ineffective and uneconomical processes [9]. Recently, Ling et al. [10] reported the decolourisation of synthetic dyes (Bromothymol Blue, Evans Blue, Fuchsin Basic, Malachite Green, Methylene Blue and Reactive Brilliant Blue R) using 0.5 U∙mL^−1^ purified Lcc from the WRF *Trametes* sp. LAC-01. Chairin et al. [11] also reported the decolourisation of synthetic dyes (Acridine Orange, Bromophenol Blue, Congo Red, Methyl Orange, Reactive Black 5 (RB5) and Remazol Brilliant Blue R) using 0.45 U∙mL^−1^ purified Lcc from the WRF *Trametes polyzona*. In a different study, decolourisation of RB5 was optimised using crude Lcc from the WRF *Trametes pubescens* with the mediator 1-hydroxybenzotriazole (HBT) [12]. The authors obtained maximum decolourisation of 150 mg∙L^−1^ RB5 (60% in 20 min) with 1.17 mM HBT and 0.5 U∙mL^−1^ Lcc. The high efficient capability of *T. pubescens* to decolourise RB5, in successive batches, when immobilised on stainless steel sponges in a fixed-bed bioreactor was also described by Enayatzamir et al. [13]. The main enzyme involved was Lcc at pH 4.5. However, a decrease in activity was reported in the final stages of the cultivation probably due to high pH values (8–9).

Textile dye wastewater is notorious for having a strong colour, with high Chemical Oxygen Demand (COD), high salt concentrations, and high pH [14]. Many studies do not take into account these peculiar parameters, which affect the activity of the degrading enzymes. Fungal Lcc activity at basic pH values are highly desirable for the decolourisation of recalcitrant dyes [5]. Therefore, the search and use of new fungal Lcc with high activity at pH > 7.0 are of particular interest [15,16,17].

Industrial effluents have variable composition, and in many cases are extremely complex. Typically, they have few nutrients when compared to laboratory culture media and, therefore, a better knowledge on the nutrients, in particular carbon and nitrogen sources required in industrial effluents for effective biodegradation, is highly desirable. Glucose is the most widely used carbon source in fungal growth media and is effective for the expression of many enzymes. However, other carbon sources can also be effective, e.g., sucrose, fructose, glycerol, and starch [18]. Myasoedova et al. [19] described the influence of carbohydrates in regulating *lcc* expression synthesised by WRF.

*Lcc* gene transcriptional regulation is influenced by several different factors (e.g., media composition and culture conditions), and this influence has been demonstrated in many WRF. In most reports, *lcc* expression is described as being regulated by several different factors, often acting synergistically [20]. In addition, the selection of microorganisms and culture media allows for the design of cost-effective processes [21,22]. According to several authors [23,24,25], expression of this enzyme can be facilitated by regulation with metal ions, lignin related aromatic compounds and derivatives, and carbon and nitrogen. In this context, the current study aims to investigate the capability of *T. versicolor* to decolourise RB5 under alkaline conditions in a fixed-bed bioreactor and evaluate the effect of different carbon sources and pH for *lcc2* expression.

## 2. Materials and Methods

### 2.1. Fungus and Medium Composition

*Trametes versicolor* MUM 04.100 was obtained from the Portuguese Culture Collection *Micoteca da Universidade do Minho* (MUM), Braga, Portugal. This strain grown on BR5 under alkaline conditions, as previously reported by Ottoni et al. [5]. The strain was maintained on Tap Water Agar-cellulose plates (15 g∙L^−1^ TWA-cellulose, Oxoid Technical Agar No. 3 (Hampshire, UK), in tap water supplemented with a Whatman grade 4 filter paper strip) and subcultured on a monthly basis. Liquid culture medium (LCM) was composed by 5.0 g∙L^−1^ sucrose, 0.5 g∙L^−1^ ammonium sulphate (Sigma, St. Louis, MO, USA), 1.7 g∙L^−1^ yeast nitrogen base (YNB) amino acids and ammonium sulphate free (Sigma), 1.0 g∙L^−1^ L-asparagine (Sigma) and 0.1 g∙L^−1^ RB5. The initial pH of the LCM was adjusted to pH 9.5, value set based on previous research works [5,6], using 1 M NaOH aqueous solution. The pre-adaption medium (PAM) consisted in LCM containing 15 g∙L^−1^ Oxoid Technical Agar No. 3.

### 2.2. Culture Conditions

#### 2.2.1. Decolourisation of RB5 in a Fixed-Bed Bioreactor

**Figure 1:**
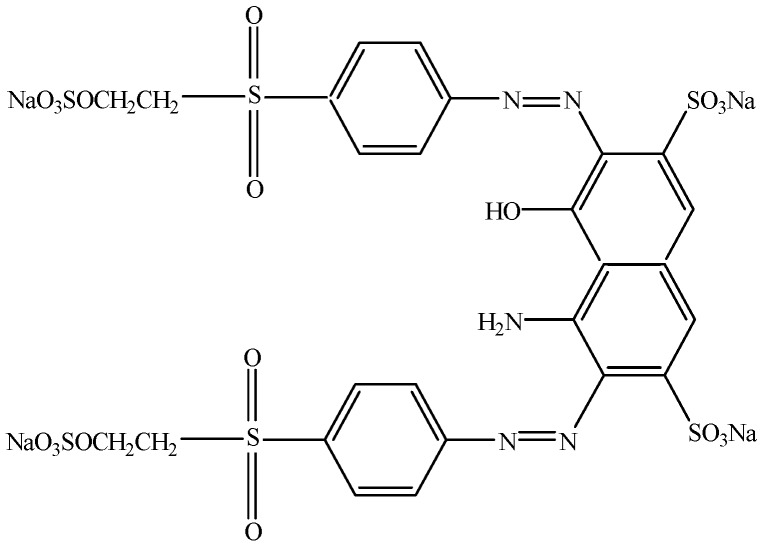
Molecular structure of Reactive Black 5 diazo compound with two N double bound N bounds.

Reactive Black 5 (RB5), also known as Remazol Black B, is a textile diazo blue dye (absorbance λ_max_ at 592 nm) with a molecular structure represented in Figure 1; a chemical formula of C_26_H_21_N_5_Na_4_O_19_S_6_ and molecular weight of 991.82 g∙mol^−1^. The Colour Index (CI) is 20505 and the Chemical Abstracts Service (CAS) numbers are 12225-25-1 and 17095-24-8. RB5 compound from Sigma-Aldrich No. 306452 (Steinheim, Germany) was used in aqueous solution.

The design of the bioreactor in this research was influenced by the design of other bioreactors and fungi used previously for Lcc enzyme production, dye decolourisation, or textile wastewater treatment [26,27,28].

**Figure 2:**
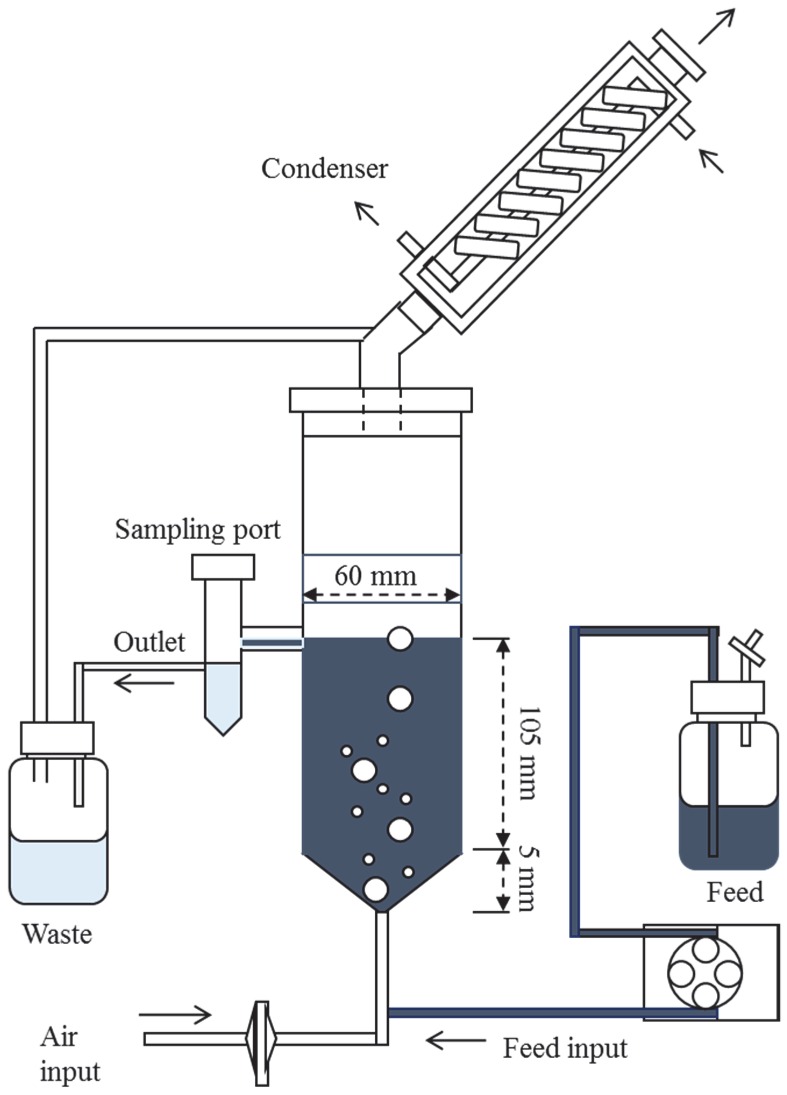
Diagram of the fixed-bed reactor used in the present study. The elements are not in scale.

Continuous RB5 decolourisation was carried out in a ca. 300 mL fixed-bed bioreactor (Figure 2) using free *T. versicolor* cells.

The bioreactor had a working volume of 260 mL and was run continuously via a peristaltic pump and with continuous agitation provided by the aeration at room temperature (ca. 30 °C) without pH control. Aeration was established by the agitation rate that avoided excessive foam. Thirteen plugs, 8 mm in diameter, cut with a sterile cork-borer from the periphery of a 7-day-old colony grown on PAM plates, were used as the inoculum. The plugs were grown in a 500 mL Erlenmeyer flask containing 260 mL of LCM and incubated for 9 days in a Certomat rotary shaker (150 rpm) at 30 °C. The biomass was vacuum-filtered and transferred into the bioreactor under aseptic conditions. The bioreactor was loaded with fresh LCM and operated in batch mode until total decolourisation. Afterwards, it started to be continuously fed with an aqueous solution of RB5 (0.1 g·L^−1^ at pH 9.5) with a mass fixed flow rate of 2.14 mg·day^−1^ and a hydraulic retention time of 7.14 days. During the time course of decolourisation when 85% or less was achieved pulses of 5 g·L^−1^ sucrose or glycerol final concentration were made at different times in order to maintain fungal metabolism for an extended fermentation time period. The continuous fermentation was run without external nitrogen source. The dye decolourisation and enzymatic activity were assessed daily.

#### 2.2.2. Carbon Source Monitoring

Sucrose concentrations were monitored by high performance liquid chromatography (Jasco AS-950, Tokyo, Japan). The detector used was a refractive index (Jasco RI-830, Tokyo, Japan). The column used was a MetaCarb 67H (300 × 6.5 mm) and its internal temperature was adjusted to 60 °C. The mobile phase used was 0.005 mol∙L^−1^ of an aqueous solution of H_2_SO_4_ at a flow rate of 0.7 mL∙min^−1^. Samples were injected in a volume of 20 µL.

In order to determine the sucrose concentrations, a standard curve was created where the initial sucrose concentration in LCM corresponded to 100%. All samples analysed were collected and monitored daily.

### 2.3. Influence of Different Carbon Sources on Expression of Lcc Gene

#### 2.3.1. Genomic DNA Isolation, Sequencing and Sequence Analysis

Five plugs, 8 mm in diameter were cut with a sterile cork-borer, from the periphery of a 7-day-old colony of TWA-cellulose and inoculated in 250 mL Erlenmeyer flasks containing 100 mL of Glucose Yeast Peptone (GYP, 3 g∙L^−1^ malt extract, 10.0 g∙L^−1^ glucose, 3 g∙L^−1^ yeast extract and 5 g∙L^−1^ peptone). These were incubated on a Certomat rotary shaker for 6 days at 30 °C and 150 rpm. Mycelium of *T. versicolor* was frozen and used for genomic DNA isolation [29]. Primers were designed, based on the sequence analysis of *lcc2* gene (GenBank accession No. Y18012.1; 5′-ATGTCGAGGTTTCACTCTCTTC-3′) [30] and the housekeeping gene β-*tubulin* (GenBank accession No. AY944858.1; 5′-CGGTGAGAGGCGTCGGACAC-3′) for *T. versicolor*. The DNA amplification was performed using a thermocycler (MyCycler™, BioRad, Hercules, CA, USA).

Reaction conditions for PCR amplification consisted of an initial denaturation at 94 °C for 2 min, 35 cycles of denaturation at 94 °C for 0.75 min, annealing at 49–60 °C (depending on the primers) for 0.5 min and extension at 72 °C for 2 min, with a final extension of 4 min at 72 °C. GoTaq DNA polymerase (Promega, Foster, CA, USA) was used in the PCR reactions. PCR products were separated by agarose gel electrophoresis and the correspondent bands for *lcc2* and β*-tubulin* purified using the (Qiagen, Hilden, Germany). DNA sequencing was conducted in both directions by Eurofins MWG Operon (Ebersberg, Germany). A homology search was conducted with the laccase sequence from *T. versicolor* using protein BLAST (Basic Local Alignment Search Tool) [31] and for comparison purposes ClustalW was used to create multiple sequence alignments [32].

#### 2.3.2. RNA Extraction and RT-PCR

Five plugs, 8 mm in diameter were cut with a sterile cork-borer from the periphery of a 7-day-old colony of *T. versicolor* grown in PAM. The plugs were inoculated in a 250 mL Erlenmeyer flask containing 100 mL GYP and incubated on a Certomat rotary shaker for 5 days at 30 °C and 150 rpm. Biomass was then retrieved and carefully washed three times with 150 mL sterile water using vacuum filtration under sterile conditions. From that biomass, ca. 1 g was transferred into 36 Erlenmeyer flasks, each containing 100 mL of the LCM (Section 2.1) with 5 g·L^−1^ of carbon source and the following conditions: Glucose, sucrose and glycerol, with pH 9.5 and without pH adjustment. The Erlenmeyer flasks were then incubated for a period of two, up to six, days at 30 °C and 150 rpm. For each condition, total RNA was extracted according to Chomczynski and Sacchi [33]. The cDNA syntheses were performed with SuperScript™ III Reverse Transcriptase 18080-093 kit (Invitrogen, Carlsbad, CA, USA). PCR amplifications were performed, as described in the previous section, with 52 °C as the optimal annealing temperature for *lcc2*. PCR products were separated by electrophoresis. Using Gel Doc XR System (Bio-Rad, Hercules, CA, USA), it was possible to visualise the gel and document images. The analysis for the quantification of expression levels of the gene of interest was performed using densitometry. The documented images of amplifications were treated with the support of the program ImageJ 1.44f [34]. Results were normalised by densitometry according to the constitutive gene expression of β-*tubulin*. Therefore, it was possible to establish the ratio between the *lcc2* gene expression relative to β*-tubulin* under different conditions, and the results are presented in arbitrary units.

### 2.4. Analytical Methods

During the experiment, the dye concentration was determined, as previously described by Ottoni et al. [5], using spectrophotometry (190 to 900 nm), and an additional control was assayed with autoclaved fungal biomass to evaluate the contribution of the fungal cell walls to dye adsorption at the maximum wavelength (λ_max_) of the RB5. The absorbance value of the LCM containing the initial concentration of dye corresponds to 100% of dye. The spectra were obtained from 5 mL of supernatant samples. pH values were recorded for each sample.

### 2.5. Enzymatic Assays

Laccase activities were determined at room temperature by oxidation of 90 µL of a 0.11 mM solution of syringaldazine (4-hydroxy-3,5-dimethoxybenzaldehyde azine, Sigma-Aldrich) in ethanol absolute (Merck, Darmstadt, Germany), 10 µL aliquots of supernatant samples, 200 µL citric acid/sodium hydrogen phosphate buffer solution (pH 6.0) mixed in a total volume of 300 µL [35]. The oxidation of syringaldazine was determined at 525 nm with an extinction coefficient ε_525 nm_ = 65,000 M^−1^·cm^−1^ using a UV/Vis spectrophotometer (Jasco 560). The same reaction mixtures, but with boiled supernatant samples, were used as blanks for each of the enzymatic activity assays. The amount of the enzyme responsible for the change of 0.01 of absorbance per minute, under the assay conditions, was defined as one unit (U) of enzyme activity; additionally, all enzyme activity values were expressed as units per litre (U·L^−1^). In order to evaluate the adsorption of the dye on the fungal mass during the reactions, the samples were collected daily and filtered according to previous studies [6,35].

## 3. Results and Discussion

### 3.1. Decolourisation of RB5

**Figure 3:**
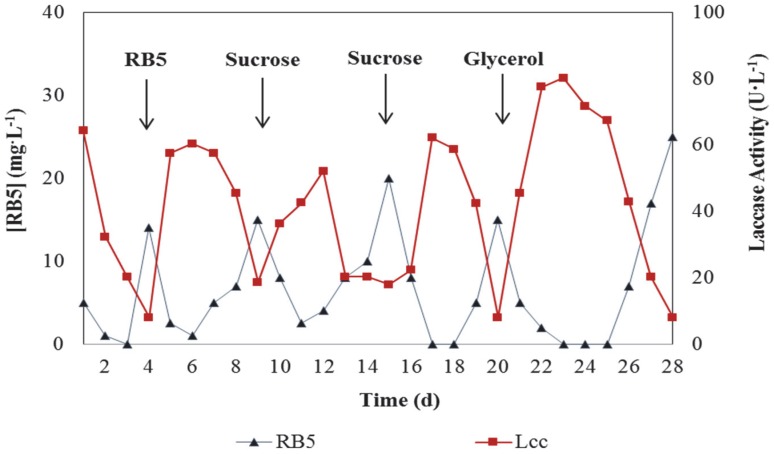
Time course of Reactive Black 5 (RB5, 0.1 g·L^−1^ at pH 9.5) concentration and Lcc activity using *T. versicolor*. The bioreactor operated in batch mode with LCM until total decolourisation and was then continuously fed with RB5 at day 4. Rows indicated when the pulses of different carbon sources at concentration of 5 g·L^−1^ were added to the RB5 solution at days 9, 15 and 20.

Maximum Lcc activities were observed on day 6 (60.4 U·L^−1^), 12 (52.1 U·L^−1^), 17 (62.2 U·L^−1^) and 23 (80.2 U·L^−1^), with a concomitant dye decolourisation of 99%, 97.5%, 100%, and 100%, respectively (Figure 3). These results show high dye decolourisation three to four days after each addition of the carbon sources. Moreover, high Lcc activity was related to the decolourisation process of RB5 and biomass growth. The nitrogen was a limited factor during the fermentation, confirming, what is generally accepted, that a high carbon-to-nitrogen ratio is required for Lcc production in WRF. The biomass purges were done at days 11, 17, and 24 to maintain the aeration and the bioreactor operational. Other enzymes were analysed (i.e., manganese peroxidase, lignin peroxidase, glyoxal oxidase, proteases, phenoloxidase), but without relevant activity (data not shown). No significant adsorption of the dye on the fungal biomass was detected.

Peaks in Lcc activity ranged from 50.9 U·L^−1^ to 80.2 U·L^−1^ depending on the carbon source used. Whenever a reduction (<85%) in the decolourisation rate was observed, a new carbon source pulse was made. When glycerol was used, the maximum value (80.2 U·L^−1^) of Lcc activity was achieved together with a decolourising rate of 100% on days 23 and 25. Kanwal and Reddy [36] evaluated the effect of different carbon sources on ligninolytic activity of *Morchella crassipes* and concluded that Lcc activity variation depended on the carbon source used. Furthermore, and in agreement with the findings on this study, glycerol was described as a good inducer for the production of Lcc using *Trametes hirsuta* [26] and *Pleurotus ostreatus* [37].

Ottoni et al. [6] used the same strain as in the present study, and extreme saline and alkaline conditions, and found an optimal Lcc activity of 119.8 U·L^−1^ was obtained for glycerol when compared to 60.0 U·L^−1^ obtained for sucrose. Rodríguez Couto et al. [26] recorded an activity of 19,394 U·L^−1^ laccase from *T. hirsta* after glycerol addition. Kachlishvili et al. [38], among seven carbon sources studied found, after glucose, glycerol as good carbon source to increasing the Lcc activity in *Cerrena unicolor*. In a co-culture process, Li et al. [39] reported to *Ganoderma lucidum* that, under the condition of glucose deprivation, the use of glycerol as a secondary carbon source produced by the yeast *Candida* sp. HSD07An leads to the overproduction of Lcc.

Most of the studies published are performed in acidic conditions despite textile effluents being alkaline. Enayatzamir et al. [13] reported the ability of *T. pubescens* to decolourise RB5 in a fixed-bed reactor at pH 4.5. Furthermore, Baccar et al. [27] found good bleaching results when using *T. versicolor* at pH 4.5 to decolourise Dycem Black TTO dye used in the tanning industry. The strain used decolourised 94% of the dye under agitation in Erlenmeyer flasks, and over 86% in a bioreactor and found that Lcc was the principal enzyme involved. On the other hand, Ottoni et al. [5,6] reported decolourisation of RB5 under alkaline conditions using the same strain as in the present study.

The decolourisation rate observed (Figure 3) was influenced by the addition of carbon sources, confirming the results of Pakshirajan and Kheria [40]. According to their data, 5.0 g·L^−1^ glucose led to optimal decolourisation of 80%. In contrast, no carbon source reduced the decolourisation to 53%. Osma et al. [2] detected maximum Lcc activities of 51 U·L^−1^, 89 U·L^−1^ and 228 U·L^−1^ using *T. pubescens*, when glucose, glycerol, and mandarin peelings, as carbon sources, respectively, were employed.

Borchert and Libra [41] analysed decolourisation rates of synthetic dyes by free cells of *T. versicolor* at pH 5.0, and suggested the possibility of reusing the cell culture for a long time without decreasing the activity of extracellular enzymes. This was confirmed by the experiments performed in this present study, where decolourisation was observed until the end of the assay (Figure 3).

### 3.2. Gene Expression of lcc2 Gene

**Figure 4:**
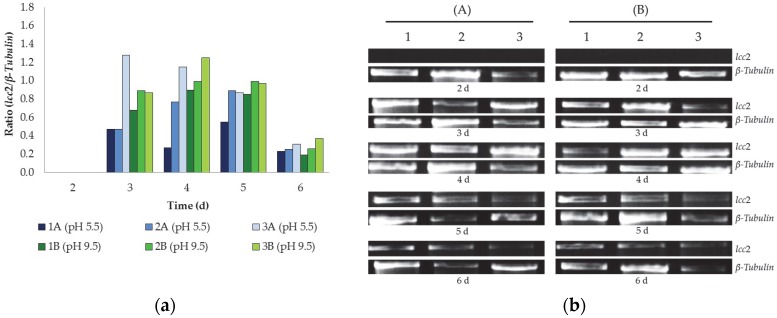
Detection of *lcc2* gene expression by the strain *T. versicolor* MUM 04.100 under different culture conditions in batch shake flasks: (**a**) by densitometry with arbitrary unites based on the (**b**) RT-PCR transcripts. (A) LCM with initial pH 5.5; (B) LCM with initial pH 9.5 and carbon sources evaluated, (1) glucose, (2) sucrose, (3) glycerol.

A variety of reports highlighting the inducing effect of carbon sources in the decolourisation of dyes are available [10,19,24,25,42]. Figure 4 shows the results obtained for the expression of *lcc2* using different carbon sources and the initial 9.5 and final 5.5 pHs obtained during the fermentation study. To perform this, oligonucleotide primers, based on existing *T. versicolor* laccase gene (GenBank accession No. Y18012.1), were used and a specific band of 1563 bp was amplified from *T. versicolor* chromosomal DNA. Sequence analysis of the products revealed 100% identity with the laccase Y18012.1 and high similarity with other *T. versicolor* laccase sequences, indicating that *T. versicolor* laccase gene was successfully cloned. The transcriptional regulation of *T. versicolor lcc2* was studied by RT-PCR analysis during growth on different carbon sources.

The *lcc2* gene was expressed at different levels in the time period between three and six days, with the highest values occurring between three and four days, with their subsequent reduction (Figure 4a). In contrast, at the 2nd day of incubation no *lcc2* gene was expressed for any conditions studied. *Trametes versicolor* MUM 04.100 showed a higher expression of this enzyme when using glycerol as a carbon source, followed by sucrose and then glucose. Glucose is not the optimal substrate to *lcc2* expression, which is corroborated by Rodríguez Couto et al. [26] for Lcc activity for *T. hirsuta* and Osma et al. [2] for *T. pubescens*.

The highest *lcc2* expression (1.28) was detected for glycerol at pH 5.5 after three days of incubation (Figure 4). However, pH 9.5 was generally more favorable than the acidic pH; however, it is known that, after three days, due a weak buffer condition when compared to the real textile effluents, the high pH can drop down to ca. 4.5–5.5, which is similar to the other pH studied. There was an abrupt reduction in the *lcc2* expression on day 6, coinciding with the reduction Lcc activity in the bioreactor (see Figure 3). According to Baldrian [43], fungal Lcc activity typically exhibits an optimum pH in acidic conditions; however, our results indicate that increased expression of the enzyme was obtained under alkaline conditions. Similar results were obtained elsewhere with *Trametes trogii* [44] and *Cerrena unicolor* [45], where Lcc was found to be stable at high pH values. Additionally, Liu et al. [46] developed a mutant Lac3T93, considered an efficient catalyst, with greatly increased enzymatic activity and decolourisation capacity under alkaline conditions. They found that the pH 8.0 for dye degradation was similar when using Lac3T93 or CotA-laccase from *Bacillus subtilis* (pH 8.0–9.0), but higher than for bacterial laccases (pH 5.0–7.5). Recently, Andriani et al. [47] described a higher *Lcc* expression by *Bjerkandera adusta* at pH 8.2. In addition, Guan et al. [48] also noticed, when testing a recombinant purified Lcc, that it was highly stable at alkaline pH and high temperatures.

The pH of the medium was only adjusted to 9.5 at the beginning of the assay employed herein and at the end of the assay the pH of the medium varied between 4.5 and 5.0. Nevertheless, *T. versicolor* MUM 04.100 was able to express *lcc2* under alkaline conditions (Figure 4). This finding corroborates previous studies [5,6], which report good Lcc performance under alkaline conditions for the same strain.

## 4. Conclusions

The present study demonstrates that using *T. versicolor* MUM 04.100 under alkaline conditions allows for the decolourisation of RB5, ranging from 80% to 100%. Glycerol was found to be an optimal carbon source for the strain, making it an attractive alternative source of *lcc2* expression and providing a good basis for further evaluation studies on alkaline conditions, and the mechanisms and metabolic intermediates that are formed from RB5.
